# Метаанализ экспериментальных исследований влияния монотерапии мелатонином на уровень тиреоидных гормонов и глюкокортикоидов у крыс, содержащихся в стандартных условиях

**DOI:** 10.14341/probl13396

**Published:** 2024-11-05

**Authors:** Н. В. Кузьменко, В. А. Цырлин, М. Г. Плисс

**Affiliations:** Национальный медицинский исследовательский центр им. В.А. Алмазова; Первый Санкт-Петербургский государственный медицинский университет им. академика И.П. Павлова; Национальный медицинский исследовательский центр им. В.А. Алмазова; Национальный медицинский исследовательский центр им. В.А. Алмазова

**Keywords:** мелатонин, щитовидная железа, надпочечники, тиреотропный гормон гипофиза, трийодтиронин, тироксин, кортикостерон

## Abstract

**ОБОСНОВАНИЕ:**

ОБОСНОВАНИЕ. Известно, что мелатонин модулирует суточные и сезонные ритмы метаболизма, размножения и поведения. Однако до сих пор неясен эффект приема экзогенного мелатонина на функционирование щитовидной железы и надпочечников у видов без четко выраженной сезонности в репродукции.

**ЦЕЛЬ ИССЛЕДОВАНИЯ:**

ЦЕЛЬ ИССЛЕДОВАНИЯ. С помощью метаанализа публикаций исследовать эффект монотерапии мелатонином на концентрацию тиреотропного гормона гипофиза, тиреоидных гормонов (ТГ), адренокортикотропного гормона гипофиза и кортикостерона (КС) у крыс, содержащихся в стандартных лабораторных условиях.

**МАТЕРИАЛЫ И МЕТОДЫ:**

МАТЕРИАЛЫ И МЕТОДЫ. С помощью программы Review Manager 5.3 мы провели метаанализ публикаций, исследующих влияние монотерапии мелатонином на функционирование щитовидной железы (22 работы) и надпочечников (20 работ) у крыс, содержащихся в стандартных условиях.

**РЕЗУЛЬТАТЫ:**

РЕЗУЛЬТАТЫ. По результатам нашего метаанализа, эффекты мелатонина на уровень ТГ и КС зависят от дозы и продолжительности терапии. Снижение ТГ и КС было ассоциировано с терапией продолжительностью не более 4–5 недель и с высокими дозами мелатонина. Увеличение КС и тенденция повышения уровня ТГ наблюдались при более длительной терапии. Однако в единичных исследованиях продемонстрировали снижение ТГ при очень длительной терапии мелатонином (≥32 недели). Среди всех ТГ максимальную чувствительность к экзогенному мелатонину показал общий тироксин (Т4), что свидетельствует о влиянии мелатонина на секреторную функцию щитовидной железы. Кроме того, мелатонин увеличивал относительную массу надпочечников. Не было получено убедительных доказательств влияния на эффекты мелатонина способа и времени введения, а также времени забора проб крови.

**ЗАКЛЮЧЕНИЕ:**

ЗАКЛЮЧЕНИЕ. В итоге экзогенный мелатонин может модулировать уровень ТГ и КС, даже у видов без четко выраженной сезонности в репродуктивной функции.

## Abstract (en)

**BACKGROUND:**

BACKGROUND: Melatonin is known to modulate circadian and seasonal rhythms in metabolism, reproduction, and behavior. However, the effect of exogenous melatonin supplementation on the functioning of the thyroid and adrenal glands in species without a clear seasonality in reproduction is still unclear.

**AIM:**

AIM: Using a meta-analysis of publications, to investigate the effect of melatonin monotherapy on the concentrations of pituitary thyroid-stimulating hormone, thyroid hormones (TG), pituitary adrenocorticotropic hormone and corticosterone (CS) in rats kept under standard laboratory conditions.

**MATERIALS AND METHODS:**

MATERIALS AND METHODS: In our work, using the Review Manager 5.3 program, we conducted a meta-analysis of publications examining the effect of melatonin monotherapy on the functioning of the thyroid gland (22 papers) and adrenal glands (20 papers) in rats kept under standard conditions.

**RESULTS:**

RESULTS: According to the results of our meta-analysis, the effects of melatonin on the levels of TG and CS depend on the dose and duration of therapy. A decrease in TG and CS was associated with therapy lasting no more than 4-5 weeks and with high doses of melatonin. An increase in CS and a trend toward increased TG levels were observed with longer therapy. However, a few studies have observed a decrease in TG with very long-term melatonin therapy (≥32 weeks). Among all TGs, total thyroxine (T4) showed maximum sensitivity to exogenous melatonin, which indicates the influence of melatonin on the secretory function of the thyroid gland. In addition, melatonin increased the relative weight of the adrenal glands. There was no convincing evidence that the effects of melatonin were influenced by the route and timing of administration, or the timing of blood sampling.

**CONCLUSION:**

CONCLUSION: As a result, exogenous melatonin can modulate TG and CS levels, even in species without a clear seasonality in reproductive function.

## ОБОСНОВАНИЕ

В настоящее время гормон эпифиза мелатонин активно исследуется на предмет использования для профилактики и терапии сердечно-сосудистых, эндокринных, нервных, психических, онкологических и других заболеваний. Широкий спектр терапевтических эффектов мелатонина (гипотензивный, кардиопротективный, улучшение показателей липидного профиля, иммунопротективный, антипролиферативный, нейропротективный, антидепрессивный и др.) объясняется тем, что чувствительные к мелатонину мембранные и ядерные рецепторы расположены практически во всех органах и тканях [1]. Изучая терапевтическое действие мелатонина, исследователи делают основной акцент на его эффекты, реализующиеся через мелатонин-чувствительные рецепторы органов-мишеней (сердца, сосудов, печени, поджелудочной железы, селезенки, тимуса и т.д.), а также на антиоксидантные свойства. При этом часто игнорируется эффект мелатонина на активность тиреоидных гормонов (ТГ) и кортикостерона (КС), которые определяют общий метаболизм.

Известно, что увеличение эндогенного мелатонина при уменьшении длины дня вызывает у позвоночных уменьшение секреции тиреотропного гормона гипофиза (ТТГ) в рars tuberalis, уменьшение активности дейодиназы 2, увеличение активности дейодиназы 3 и снижение уровня активного трийодтиронина (Т3) [2]. Несмотря на то, что уровень глюкокортикоидов также имеет четкий суточный и сезонный профиль, до сих пор нет однозначных представлений о механизмах регуляции секреции гормонов коры надпочечников с помощью мелатонина. Установлено, что супрахиазматическое ядро гипоталамуса контролирует циркадную секрецию глюкокортикоидов через моносинаптические проекции в паравентрикулярное ядро гипоталамуса, регулируя уровень кортикотропин-рилизинг гормона, а также через полисинаптический путь к надпочечникам [3][4]. В работе [4] было продемонстрировано вызванное светом быстрое снижение уровня кортикостерона в плазме, которое не было связано с уменьшением уровня адренокортикотропного гормона гипофиза (АКТГ). Есть предположение, что эффект АКТГ на кору надпочечников будет определяться сезоном и уровнем половых гормонов. Также есть мнение, что масса надпочечников зависит от сезона [5]. В некоторых исследованиях была обнаружена взаимосвязь между уровнем тиролиберина и концентрацией АКТГ [6]. В структурах головного мозга, ассоциированных с биологическими ритмами (супрахиазматическое ядро, pars tuberalis гипофиза), а также в надпочечниках представлены в основном мелатонинергические рецепторы 1 типа (МТ1) [7-9]. При этом в щитовидной железе (ЩЖ) были идентифицированы МТ1 и МТ2 рецепторы [10].

Исследования показали, что дефицит эндогенного мелатонина оказывает влияние на уровень ТГ и КС. Так, после удаления пинеальной железы у крыс наблюдалось увеличение уровня ТТГ, свободного тироксина (свТ4) и свободного трийодтиронина (свТ3) без существенных изменений общего Т4 и Т3 [11][12]. Постоянное освещение было ассоциировано со снижением Т4 у самцов, но не у самок [13]. У крыс, содержащихся при длинном дне, в среднем уровень ТГ и КС был выше, чем у животных, находящихся в условиях короткого дня или стандартного освещения [14]. Однако есть наблюдения, что постоянное освещение снижает средний уровень КС, поскольку полностью нивелирует акрофазу [15]. Пинеалэктомия вызвала у крыс увеличение среднего уровня АКТГ и КС, а также изменение циркадного профиля КС [12][16][17]. В противоположность, в исследовании [18] не было зафиксировано каких-либо изменений в суточном ритме и концентрации мелатонина и КС после пинеалэктомии. По результатам одних исследований, пинеалэктомия увеличивала массу надпочечников [19], по результатам других — не изменяла массу надпочечников, но увеличивала массу ЩЖ [20]. Введение экзогенного мелатонина нивелировало эффекты пинеалэктомии на уровень свТ3 и свТ4, но не КС [12]. В условиях постоянного освещения мелатонин незначительно снижал уровень Т3, но не Т4 [13].

## ЦЕЛЬ ИССЛЕДОВАНИЯ

Цель нашей работы — исследовать с помощью метаанализа публикаций эффект монотерапии мелатонином на концентрацию ТТГ, ТГ, АКТГ и КС у крыс без патологий, содержащихся в стандартных (или близких к стандартным) лабораторных условиях. Экспериментальные исследования действия мелатонина на параметры общего метаболизма у крыс интересны в том числе по причине того, что репродуктивная функция крыс, как и у людей, не имеет четко выраженной сезонности.

## МАТЕРИАЛЫ И МЕТОДЫ

Метаанализ был выполнен в соответствии с рекомендациями PRISMA (http://www.prisma-statement.org). Поиск исследований осуществлялся весной-летом 2023 г. на английском и русском языках независимо двумя людьми в базах PubMed, Scopus, Google Scholar, elibrary без ограничения периода публикации. При поиске были использованы ключевые слова, связанные с ТГ и глюкокортикоидами (щитовидная железа, надпочечники, тиреотропный гормон гипофиза, тиролиберин, трийодтиронин, тироксин, адренокортикотропный гормон гипофиза, кортикотропин-рилизинг гормон, кортикостерон, кортизол), которые сочетались с воздействием (мелатонин, терапия мелатонином, инфузия мелатонина), объектом исследования (крысы). Кроме того, дополнительно были просмотрены списки литературы публикаций, отобранных для метаанализа.

В метаанализ были включены исследования только монотерапии мелатонином. Отбирались работы, в которых животные содержались в стандартных лабораторных условиях или условиях, близких к стандартным. Были исключены исследования, в которых крысы находились при постоянных или непропорционально длительных темноте или освещении. Если в публикации не были уточнены условия содержания, то мы считали, что животные содержались в стандартных лабораторных условиях. Также мы исключили экспериментальные работы, поставленные на линиях крыс, чувствительных к изменению фотопериода (например Fisher) и с генетическими нарушениями метаболизма (например Zucker). В метаанализ включались исследования, проведенные только на интактных или ложнооперированных животных. Исключались работы с использованием крысят до 1,5 месяца возраста и беременных самок. Время введения мелатонина и регистрации показателей также имели значения для отбора работ, в которых мелатонин вводился однократно. В этом случае были отобраны только те исследования, в которых после однократного введения мелатонина регистрация показателей была осуществлена не позднее 60 мин после введения гормона. В статистический анализ не были включены работы, исследующие эффекты мелатонина при центральном введении. Кроме того, были исключены публикации, в которых результаты были отображены в непонятной форме, не позволяющей оценить среднее значение и SD / SEM.

Из отобранных работ извлекались данные по уровню в крови ТТГ, Т3, Т4, свТ3, свТ4, КС, массе щитовидной железы и надпочечников (абсолютной и относительной). После извлечения из публикаций значения исследуемых биохимических показателей были переведены в одинаковые единицы измерения с помощью online калькуляторов http://unitslab.com/ru и http://www.scymed.com/en/smnxtm/tmbbcbb1.htm : мМЕ/л — для ТТГ, нг/мл — для Т3, КС и АКТГ, мкг/дл — для Т4, нг/дл — для свТ3 и свТ4. Для перевода ТТГ использовали стандарт Интернациональная Единица — 36,6 мг [21], для АКТГ — стандарт 1 мкг/мл эквивалентен 0,014 мМЕ/100 мл [22].

Данные объединялись в субгруппы по времени терапии мелатонином (без учета дозы). Кроме того, там, где позволяло количество исследований, был проведен анализ зависимости эффекта от дозы, способа и времени введения мелатонина, времени забора крови, возраста животных.

Метаанализ результатов исследований был проведен с помощью статистической программы Review Manager 5.3 (Cochrane Library). Для анализа был использован inverse variance тест (Mean Difference). Гетерогенность включенных в метаанализ исследований устанавливали по критерию I2. Выбор модели фиксированных или рандомизированных эффектов осуществляли в соответствии с рекомендациями [23]. Для оценки статистической значимости суммарных результатов применяли Z-тест. Предвзятость при отборе публикаций проверяли с помощью графика-воронки. Доверительный интервал — 95%. Различия считались статистически значимыми при р<0,05. В тексте данные приведены в виде медианы и интерквартильного размаха или в виде среднего значения ± стандартного отклонения.

## РЕЗУЛЬТАТЫ И ИХ ОБСУЖДЕНИЕ

В базах было найдено 149 работ (из них 19 обзоров), изучающих влияние мелатонина на уровень ТТГ и ТГ у крыс, и 195 работ (из них 16 обзоров), исследующих влияние мелатонина на гормоны коры надпочечников. После исключения публикаций по заголовкам и резюме 39 публикаций были отобраны для метаанализа [12][13][24–60] (табл. 1).

## Влияние мелатонина на уровень ТТГ и тиреоидных гормонов

Для метаанализа были отобраны 22 публикации, исследующие влияния монотерапии мелатонином на уровень циркулирующего ТТГ и ТГ: ТТГ — 12 работ, Т3 — 16 работ, Т4 — 15 работ, 4 — свТ3 и свТ4 (табл. 1). Графики воронки не показали предвзятости при отборе публикаций. Два исследования [50][55] не были включены в количественный статистический анализ по причине содержания крыс в условиях короткого дня (8/16) и естественного освещения. В двух работах исследовалось влияние однократного введения мелатонина на уровень гормонов, в остальных мелатонин вводился от 4 дней до 80 недель. В исследованиях был использован мелатонин в дозах от 0,05 до 10 мг/кг/день, который вводился подкожно, внутрибрюшинно или орально (с питьевой водой или через зонд). В 4 работах сообщается о сборе проб крови натощак (табл. 1).

Однократное внутривенное введение мелатонина не изменяло уровень Т3 и Т4, но уменьшало уровень ТТГ (рис. 1). Исследование [29] продемонстрировало дозозависимый эффект мелатонина на уровень ТТГ, но из-за отсутствия других публикаций, подтверждающих это, сделать окончательный вывод нельзя.

Поскольку результаты работ очень гетерогенны, не было получено убедительных данных об изменении уровня ТТГ и ТГ после продолжительной терапии мелатонином (рис. 2), но наблюдалась тенденция снижения ТТГ, Т3 и Т4 при более высоких дозах мелатонина (табл. 2). Начало терапии было ассоциировано со снижением уровня Т4 в среднем на -0,82 [ -1,45, -0,19] мкг/дл (I²=93%, Z=2,54, р=0,01), однако при терапии более 7 недель отмечалось незначительное увеличение Т4 в среднем на 0,56 [ -0,07, 1,20] мкг/дл (I²=83%, Z=1,73, р=0,08). Тенденции для Т3 были противоположны Т4 через 4–5 дней терапии, но если терапии длилась дольше, то Т3 и Т4 изменялись однонаправленно. В одном исследовании наблюдали снижение ТГ при очень длительной терапии мелатонином (≥32 недели) [58] (рис. 2). Метаанализ не выявил взаимосвязи между способом введения мелатонина и эффектом терапии (табл. 2).

Эффект мелатонина на ТТГ и ТГ не был очевиден ни при дневном, ни при ночном заборе крови (рис. 3). Известно, что в течение суток у крыс, содержащихся в условиях 12 день: 12 ночь, уровень мелатонина максимален ночью и минимален днем, ТТГ максимален вечером и минимален утром, Т3 и Т4 максимальны утром и минимальны вечером [47]. Следует отметить, что введение мелатонина даже в малых дозах (0,5 и 1 мг/кг/день) существенно увеличивало уровень циркулирующего мелатонина в течение всего дня [13][47]. Введение мелатонина перед темной фазой (17:00–18:30) смещало циркадные профили Т3 и Т4, но не мелатонина и ТТГ [47]. Однако при использовании аналогичных доз и времени введения мелатонина другие авторы не наблюдали изменения циркадного профиля свТ3 и свТ4 [12].

Молодой возраст животных был ассоциирован со значительным снижением Т4 при терапии мелатонином (без учета продолжительности) (табл. 2). Через 7–10 дней терапии у всех животных, кроме старых, отмечалось снижение уровня Т3 в среднем на -0,11 [ -0,18, -0,04] нг/мл (I²=55%, Z=2,96, р=0,003), а Т4 на -0,68 [ -1,25, -0,11] мкг/дл (I²=89%, Z=2,34, р=0,02). Показано, что у крыс максимальная активность ТГ наблюдается в 1,5–2-месячном возрасте, к 3 месяцам резко снижается и далее постепенно уменьшается [61]. У 24-месячных крыс по сравнению с 6-месячными уровень Т3 снижен в 3,4 раза, Т4 — в 2 раза, свТ3 — в 2 раза, свТ4 — в 4,6 раза, без существенных изменений ТТГ [62], что указывает на уменьшение секреторной активности ЩЖ у старых животных. По данным работы [38], 10-дневная терапия мелатонином восстанавливала функцию ЩЖ и дозозависимо повышала уровень Т3 и Т4 у 19-месячных крыс.

В работе [13] установили, что мелатонин после 4-недельной терапии (2 мг/кг/день, подкожно) снижает уровень Т4 у самцов и увеличивает Т4 у самок. В работе [50] также наблюдали у самок увеличение Т3 и Т4 после 7 недель подкожного введения мелатонина в дозе 1 мг/кг/день, однако в этом исследовании крысы содержались в условиях короткого дня (8/16).

При содержании животных в условиях естественного освещения введение мелатонина в течение 10 дней в дозе 1 мг/кг/день снижало уровень Т3 и зимой и летом, а уровень Т4 и ТТГ — только зимой [55]. Можно предположить, что преобладающий эффект экзогенного мелатонина заключается в угнетении превращения Т4 в Т3. На это также указывает и то, что в условиях постоянного освещения мелатонин незначительно снижал уровень Т3, но не Т4 [13]. Было установлено, что мелатонин может увеличивать экспрессию DIO3, уменьшая активность ДНК-метилтрансферазы [63]. Однако наш метаанализ показал высокую чувствительность Т4 к терапии мелатонином, что свидетельствует о влиянии экзогенного мелатонина на секреторную функцию ЩЖ. В исследованиях in vitro было обнаружено, что мелатонин снижает уровень цАМФ в эксплантатах щитовидной железы [64], а также дозозависимо уменьшает митотическую активность фолликулярных клеток ЩЖ [65][66]. В работе [67] у крыс Wistar после 4-недельной терапии мелатонином (5 мг/кг/день) наблюдали подавление функциональной активности ЩЖ — уменьшение площади фолликулярного, интерфолликулярного эпителия и высоты тироцитов, увеличение накопления коллоида и соединительной ткани. По данным одних работ, после терапии масса ЩЖ уменьшается [41, 66], по данным других — не изменяется [68].

**Table:** Таблица 1. Публикации, отобранные для метаанализа Примечание: ТТГ — тиреотропный гормон гипофиза, Т3 — общий трийодтиронин, свТ3 — свободный трийодтиронин, Т4 — тироксин, свТ4 — свободный тироксин, АКТГ — адренокортикотропный гормон гипофиза, МЩ — масса щитовидной железы, МН — масса надпочечников, * — есть сообщение о рандомизации, # — есть сообщение о заборе проб крови натощак, в.в. — внутривенное введение, в.б. — внутрибрюшинное введение, п.к. — подкожное введение, п.о. — пероральное введение.

Однократное введение
Arushanian E.B.,1994 [24]	0,1, 1, 10 мг/кг, в.б., 30 мин	естеств.	Белые лаб.	взрос.	8 / 8	КС	-
Barchas J.,1969 [25]	1 мг/кг, п.к., 30 мин	12 / 12	Long-Evans	2,5	10 / 10*	КС	-
Gromova E.A.,1967 [26]	0,5, 1, 2 мг/кг, п.к., 60 мин	12 / 12	Wistar	взрос.	4–6 / 12	КС	-
Maslova L.N.,1973 (1) [27]	4 мг/кг, п.к., 60 мин	-	Белые лаб.	взрос.	10 / 6	КС	-
Maslova L.N.,1973 (2) [27]	2 мг/кг, в.б., 60 мин	-	Белые лаб.	взрос.	5 / 5	КС	-
Maslova L.N.,1973 (2) [27]	4 мг/кг, в.б., 60 мин	-	Белые лаб.	взрос.	6 / 6	КС	-
Mattila J.,1981 [28]	1 и 10 мг/кг, в.в., 30 мин	12 / 12	Sp. Dawley	2	5 / 5	ТТГ, Т3, Т4	12:00–13:00
Mitsuma T.,1985 [29]	1, 1,25, 2,5, 5 мг/кг, в.в., 30 мин	12 / 12	Wistar	взрос.	7 / 7	ТТГ	-
Niles L.P.,1983 [30]	0,1 мг/кг, в.б., 60 мин	12 / 12	Sp. Dawley	3	8 / 8	КС	12:00
Длительная терапия
Abd Allah E.S.H.,2018 [31]	10 мг/кг/день, в.б., 6 нед	12 / 12	Wistar	взрос.	6 / 6	КС	8:30–10:00
Ahmed H.H.,2005 [32]	5 мг/кг/день, в.б., 10 дней	12 / 12	Sp. Dawley	взрос.	6 / 6*	Т3, Т4#	-
Aoyama H.,1987 [33]	5 мг/крыса/день (20 мг/кг/день), в.б., 11 нед	-	Wistar	2	6 / 6	МН	-
Arushanian E.B.,1994 [24]	0,1, 1, 10 мг/кг, в.б., 12 дней	естеств.	Белые лаб.	взрос.	8 / 8	КС	-
Baltaci A.K.,2004 [34]	3 мг/кг/день, в.б., 4 нед	12 / 12	Sp. Dawley	взрос.	10 / 10	ТТГ, Т3, Т4, свТ3, свТ4	-
Barchas J.,1969 [25]	1 мг/кг/день, п.к., 10 дней	12 / 12	Long-Evans	2,5	10 / 10*	КС, АКТГ	-
Benova T.,2013 [35]	5 мг/кг/день (40 мкг/мл), п.о. с водой (ночью), 5 нед	-	Wistar	5	12 / 12	Т3, Т4#	8:00–9:00
Bojková B.,2006 (1) [36]	0,5 мг/кг/день (4 мкг/мл), п.о. с водой (15:00–8:00), 12 нед	12 / 12	Wistarсамцы	1,5	14 / 14	КС, МН#	-
Bojková B.,2006 (2) [36]	0,5 мг/кг/день, п.о. с водой (15:00–8:00), 12 нед	12 / 12	Wistarсамки	1,5	14 / 14	КС, МН#	-
Bojková B.,2008 (1) [37]	0,5 мг/кг/день (4 мкг/мл), п.о. с водой (15:00–8:00), 11 нед	12 / 12	Sp. Dawleyсамцы	6	8 / 12	КС, МН#	-
Bojková B.,2008 (2) [37]	0,5 мг/кг/день, п.о. с водой (15:00–8:00), 11 нед	12 / 12	Sp. Dawleyсамки	6	10 / 7	КС, МН#	-
Bondarenko L.A.,2009 (1) [38]	0,05 мг/кг/день, в.б. (перед темной фазой), 10 дней	12 / 12	Wistar	19	12 / 12	Т3, Т4, ТТГ	-
Bondarenko L.A.,2009 (2) [38]	0,5 мг/кг/день, в.б. (перед темной фазой), 10 дней	12 / 12	Wistar	19	13 / 12	Т3, Т4, ТТГ	-
Brazão V.,2020 (1) [39]	5 мг/кг/день, п.о., 9 дней	12 / 12	Wistar	1,5	5 / 5	КС	-
Brazão V.,2020 (2) [39]	5 мг/кг/день, п.о., 9 дней	12 / 12	Wistar	18	5 / 5	КС	-
Esquifino A.,1997 (1) [40]	0,125 мг/кг/день (25 мкг/крыса), п.к. (перед темной фазой), 4 дня	12 / 12	Wistar	2	6 / 6	Т3, Т4, ТТГ#	-
Esquifino A.,1997 (2) [40]	0,250 мг/кг/день (50 мкг/крыса), п.к. (перед темной фазой), 4 дня	12 / 12	Wistar	2	6 / 6	Т3, Т4, ТТГ#	-
Esquifino A.,1997 (3) [40]	0,5 мг/кг/день (100 мкг/крыса), п.к. (перед темной фазой), 4 дня	12 / 12	Wistar	2	6 / 6	Т3, Т4, ТТГ#	-
Gevorkyan A.R., (1) [41]	0,05 мг/кг/день, в.б. (перед темной фазой), 10 дней	-	Wistar	3	10 / 10	Т3, Т4, МЩ	днем и ночью
Gevorkyan A.R., (2) [41]	0,5 мг/кг/день, в.б.(перед темной фазой), 10 дней	-	Wistar	3	10 / 10	Т3, Т4	днем и ночью
Gomaa A.M.,2017 [42]	10 мг/кг/день, в.б.(17:00), 6 нед	12 / 12	Wistar	взрос.	7 / 7 *	КС, МН #	-
Jiménez-Ortega V.,2012 [43]	0,5 мг/кг/день (3 мкг/мл), п.о. с водой, 4 нед	12 / 12	Wistar	1,5	45 / 45	ТТГ, КС	в течение суток
Kinson G.A.,1973 [44]	20 мг п.к. (3,5 мг/кг/день), 4 нед	12 / 12	Sp. Dawley	2	6 / 6	КС#	-
Konakchieva R.,1998 [45]	0,08 мг/кг/день, п.к. (19:00), 1 нед	12 / 12	Wistar	взрос.	5 / 5	КС	в течение суток
Mercau M.E.,2019 [46]	20 мг помпа п.к., 4,5 мг/кг/день, 3 нед	12 / 12	Wistar	взрос.	10 / 10	КС, АКТГ	9:00–10:00
Mirunalini S.,2005 (1) [47]	0,5 мг/кг/день, в.б. (17:30–18:00), 45 дней	12 / 12	Wistar	взрос.	6 / 6*	ТТГ, Т3, Т4	в течение суток
Mirunalini S.,2005 (2) [47]	1 мг/кг/день, в.б. (17:30–18:00), 45 дней	12 / 12	Wistar	взрос.	6 / 6*	ТТГ, Т3, Т4	в течение суток
Mustonen A.M.,2002 (1) [13]	п.к.помпа (12 мг, 2 мг/кг/день), 4 нед	12 / 12	Wistarсамцы	2	10 / 10*	Т3, Т4	10:00–14:00
Mustonen A.M.,2002 (2) [13]	п.к. помпа (12 мг, 2 мг/кг/день), 4 нед	12 / 12	Wistarсамки	2	10 / 10*	Т3, Т4	10:00–14:00
Nasiraei-Moghadam S.N., 2014 [48]	5 мг/кг/день, в.б., 2 нед	12 / 12	Лабор.	взрос.	7 / 7	КС	-
Nir I.,1978 [49]	100 мкг/100г/день (1 мг/кг/день), в.б. утром, 10 дней	12 / 12	Лабор.	взрос.	36 / 37	Т3, Т4	10:00–12:00
Nordio M.,1989 [50]	100 мкг/100г/день (1 мг/кг/день), п.к. (17:00), 7 нед	8 / 16	Sp. Dawleyсамки	1–2	10 / 10	Т3, Т4, МЩ#	9:00–12:00
Olukole S.G.,2019 [51]	10 мг/кг/день, в.б., 2 нед	12 / 12	Wistar	4	7 / 7	КС, АКТГ, МН	-
Ostrowska Z.,2003 [12]	50 мкг/100г/день (0.5 мг/кг/день), в.б. (17:00–18:00), 4 нед	12 / 12	Wistar	взрос.	48 / 48	свТ3, свТ4, КС	с 08:00 в течение дня
Ozturk G.,2000 [52]	10 мг/кг/день, п.к., за 2 часа до темной фазы, 1 нед	12 / 12	Sp. Dawley	2	12 / 12*	ТТГ, Т3, Т4	10:00 и 02:00
Poliandri A.H.,2006 [53]	3 мкг/мл (0,3 мг/кг/день), п.о. с водой, 1 мес	12 / 12	Wistar	2	6 / 6	ТТГ	09:00 и 01:00
Rasmussen D.D.,1999 [54]	4 мкг/мл (0,3 мг/кг/день), п.о. с водой, 48 нед	14 / 10	Sp. Dawley	10	8 / 8	Т3, Т4, КС	-
Rom-Bugoslavskaia E.S., 1986 (1) [55]	100 мкг/100г/день (1 мг/кг/день), в.б. (17:00–18:00), 10 дней	естеств.зима	Wistar	взрос.	18 / 16	ТТГ, Т3, Т4	-
Rom-Bugoslavskaia E.S., 1986 (2) [55]	100 мкг/100г/день (1 мг/кг/день), в.б. (17:00–18:00), 10 дней	естеств.лето	Wistar	взрос.	13 / 9	ТТГ, Т3, Т4	-
Sewerynek E.,1999 [56]	5 мг/кг/день, в.б.(15:00), 7 дней	14 / 10	Wistar	взрос.	9 / 9	свТ3, свТ4	-
Vaughan M.K.,1988 [57]	25 мкг/крыса/день (0,1 мг/кг/день), п.к. (16:00-18:00), 12 нед	14 / 10	Sp. Dawley	2–3	6 / 6	ТТГ, Т3, Т4	9:00–12:00
Vinogradova I.A.,2009 [58]	1,5 мг/кг/день (10 мг/л), п.о. с водой (ночью), 20 мес	12 / 12	Лабор.	4	16 / 16	ТТГ, свТ3, свТ4	-
Wang L.,2021 [59]	10 мг/кг/день, через зонд (в 16:00), 8 нед	12 / 12	Sp. Dawley	2	10 / 10*	Т3	-
Wolden-Hanson T.,2000 [60]	0,04 мг/кг/день (0,4 мкг/мл), п.о. с водой, 12 нед	14 / 10	Sp. Dawley	10	19 / 18	КС, МН	день и ночь

**Figure:**
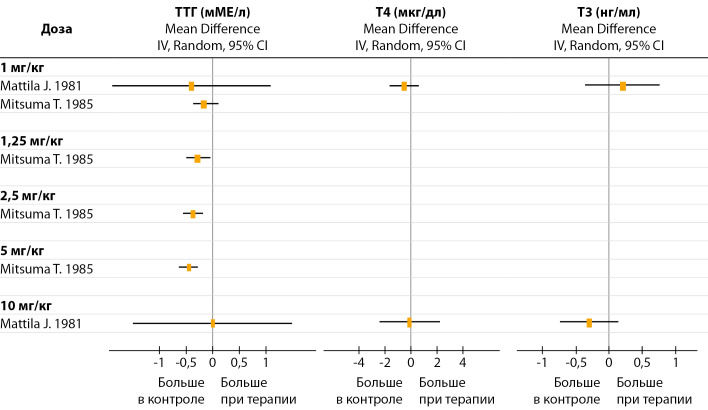
Рисунок 1. Влияние однократного внутривенного введения мелатонина на уровень ТТГ и тиреоидных гормонов.

**Figure:**
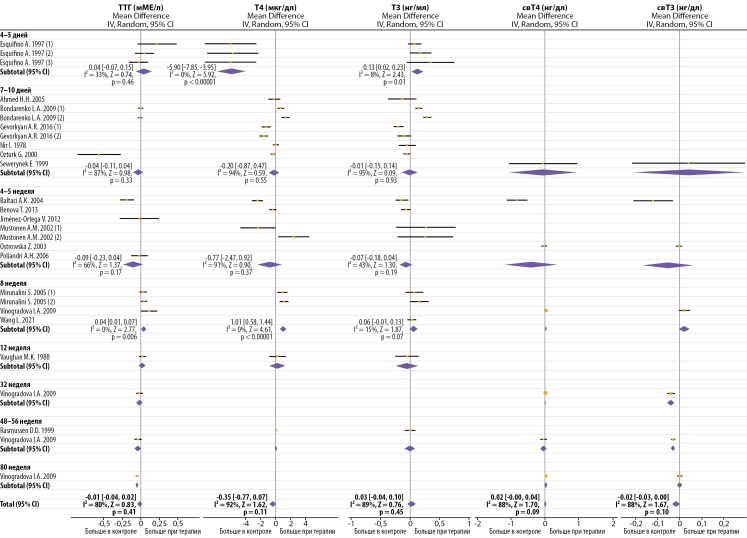
Рисунок 2. Влияние продолжительной терапии мелатонином на уровень циркулирующих ТТГ и тиреоидных гормонов. Без дополнительной стандартизации по дозе и способу введения.

**Figure:**
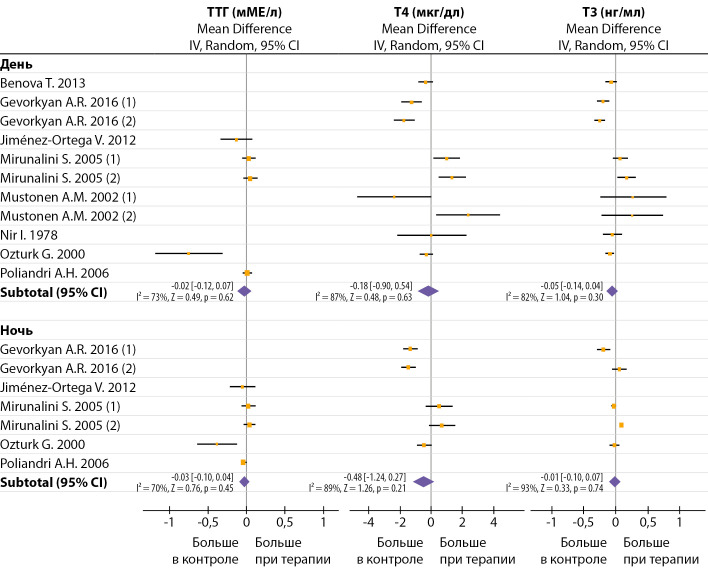
Рисунок 3. Влияние продолжительной терапии мелатонином на изменение дневных и ночных значений ТТГ и тиреоидных гормонов. Без дополнительной стандартизации по дозе и способу введения.

## Влияние мелатонина на уровень кортикостерона

Для метаанализа было отобрано 20 публикаций, исследующих влияние монотерапии мелатонином на гормоны коры надпочечников крыс. В 5 работах оценивали влияние однократного введения мелатонина, в 16 — длительного (от 1 до 40 недель). Кроме того, в 3 работах исследовали изменение циркулирующего АКТГ после 1–3 недель введения мелатонина и в 6 работах — массу надпочечников после 1–12-недельной терапии мелатонином (табл. 1). Мелатонин вводился подкожно, внутрибрюшинно и орально (в основном с питьевой водой). В 4 работах сообщается о сборе проб крови натощак (табл. 1).

Для однократного введения авторы использовали мелатонин в дозах 0,1–10 мг/кг, который вводили подкожно или внутрибрюшинно. Через 30–60 мин после введения мелатонина в дозе ≥1 мг/кг в большинстве работ наблюдали повышение уровня КС. В меньших дозах мелатонин не оказывал значимого влияния на уровень КС (рис. 4). По результатам одной работы [25], однократное подкожное введение мелатонина в дозе 1 мг/кг не изменяло уровень АКТГ (рис. 4). Интрацеребровентрикулярная инфузия мелатонина вызывала у крыс существенное снижение циркулирующих АКТГ, но не КС [69]. По данным другого исследования [27], центральное введение мелатонина существенно увеличивало уровень КС в крови. Эксперименты in vitro показали, что при обработке изолированных клеток коры надпочечников самок крыс мелатонином секреция КС уменьшается при низких дозах и не изменяется при высоких дозах; при этом у самцов мелатонин вызывал дозозависимое увеличение КС. Ни у самцов, ни у самок мелатонин (независимо от дозы) не изменял стимулирующий эффект АКТГ на секрецию КС [70]. Однако в другом исследовании [8] после обработки АКТГ мелатонин снижал секрецию КС вечером, но не утром. В работе [30] наблюдали снижение КС через 1 час после введения мелатонина ночью, днем же аналогичная инъекция гормона не влияла на уровень КС. Эксперименты [71] показали, что активность эфферентных надпочечниковых нервов уменьшается при низких дозах и увеличивается при высоких дозах мелатонина.

Для длительной терапии авторы использовали мелатонин в дозах от 0,04 до 10 мг/кг/день. После 1–4 недель терапии большинство авторов наблюдали существенное снижение уровня КС, в среднем на -78,53 [ -112,65, -44,40] нг/мл, I²=96%, Z=4,51, P<0,00001. Однако после 11–40 недель терапии уровень КС существенно увеличивался, в среднем на 51,83 [17,88, 85,78] нг/мл, I²=78%, Z=2,99, P=0,003 (рис. 5). Оценка зависимости эффекта терапии от дозы показала, что в дозе ≤1 мг/кг/день мелатонин существенно не изменяет уровень КС, а в дозе 3,5–10 мг/кг/день значительно понижает КС (табл. 2). Однако по результатам работы [24], мелатонин, вводимый в течение 12 дней в дозах 0,1, 1 и 10 мг/кг/день, дозозависимо повышал уровень КС (данные не включены в статистический анализ, т.к. представлены в относительных единицах). Наш метаанализ показал, что способ введения мелатонина не оказывал существенного влияния на уровень КС (табл. 2).

Данные влияния мелатонина на уровень КС в зависимости от времени забора проб крови очень противоречивы. Одни исследователи наблюдали после терапии увеличение КС днем и уменьшение КС ночью, другие исследователи получили противоположные результаты (рис. 6). В течение суток у крыс минимальный уровень КС отмечается утром, максимальный — вечером. Введение мелатонина в дозе 0,5 мг/кг/день перед темной фазой (17:00–18:00) смещало акрофазу КС на утро, а надир на вечер [12], но более низкие дозы мелатонина и более позднее введение (19:00) не изменяли циркадный профиль КС [45].

По результатам метаанализа, у старых крыс снижение КС после терапии мелатонином было незначительно больше, чем у молодых животных (табл. 2). Известно, что при старении уровень КС увеличивается [39][72], и при одновременном исследовании 1,5-месячных и 18-месячных крыс 9-дневная терапия мелатонином (в дозе 5 мг/кг/день, орально) эффективнее снижала КС у старых животных [39].

По данным [25][51], 10–14 дней терапии мелатонином в дозах 1 и 10 мг/кг/день не оказывали влияния на уровень циркулирующего АКТГ, однако после 3 недель терапии в дозе 4,5 мг/кг/день исследователи [46] наблюдали увеличение АКТГ в крови (рис. 5). Также есть наблюдения, что 10-дневное введение мелатонина в дозе 1 мг/кг/день существенно не изменяло уровень АКТГ в гипофизе [25]. 6-дневное введение мелатонина (1,25 мг/мл) снижало аффинность глюкокортикоидных рецепторов в гипоталамусе, гипофизе и гиппокампе [73].

Терапия мелатонином не влияла на абсолютный вес надпочечников, но значительно увеличивала их относительную массу как при низких [36][37], так и при более высоких дозах гормона (рис. 5) [51]. Центральное введение мелатонина в течение 10 дней также увеличивало массу надпочечников [74].

Два исследования [36][37] не выявили различий у самцов и самок в изменении КС и относительной массы надпочечников после терапии мелатонином в течение 11–12 недель.

**Figure:**
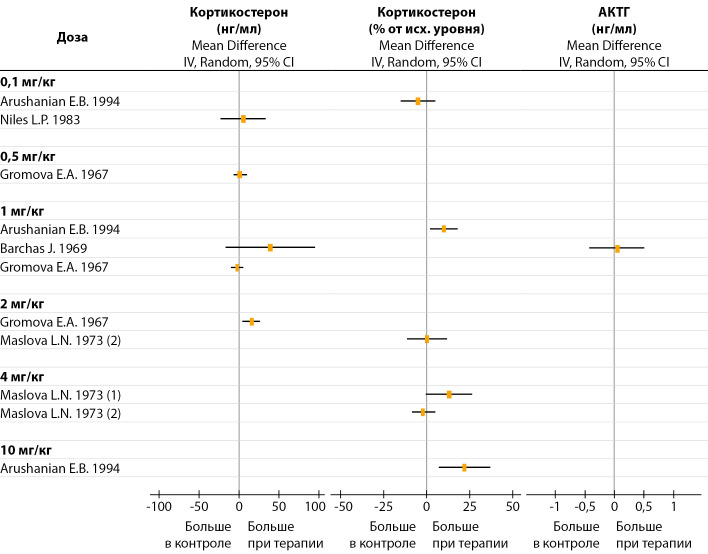
Рисунок 4. Влияние однократного введения мелатонина на уровень кортикостерона и АКТГ.

**Table:** Таблица 2. Зависимость эффекта продолжительной терапии мелатонином на уровень тиреоидных гормонов и кортикостерона от дозы, способа, времени введения и возраста крыс

	Времянед.	Дозамг/кг/день	Nработ	Объем выборкитерапия / контроль	Средняя разность	I2%	Z	P
Т3 (нг/мл)
≤1 мг/кг/день	1–48	0,05–1	7	121 / 119	0,06 [ -0,04, 0,16]	90	1,18	0,24
≥2 мг/кг/день	1–8	2–10	6	70 / 70	-0,05 [ -0,11, 0,01]	50	1,56	0,12
подкожно	1–12	0,125–10	4	56 / 56	0,07 [ -0,04, 0,18]	61	1,24	0,22
внутрибрюшинно	1–8	0,05–5	6	105 / 103	0,01 [ -0,12, 0,14]	94	0,14	0,89
орально	5–48	0,3–10	3	30 / 30	-0,01 [ -0,07, 0,05]	44	0,36	0,72
перед и в темную фазу	1–12	0,05–10	8	115 / 114	0,05 [ -0,04, 0,14]	91	1,09	0,28
только молодые крысы (2–3 мес)	1–12	0,05–10	6	86 / 86	0,00 [ -0,08, 0,08]	75	0,02	0,98
Т4 (мкг/дл)
≤1 мг/кг/день	1–48	0,05–1	7	125 / 125	-0,23 [ -0,78, 0,31]	93	0,84	0,40
≥2 мг/кг/день	1–5	2–10	5	60 / 60	-0,63 [ -1,60, 0,33]	87	1,28	0,20
подкожно	1–12	0,125–10	4	56 / 56	-1,86 [ -3,43, -0,30]	86	2,33	0,02
внутрибрюшинно	1–8	0,05–5	6	109 / 109	-0,17 [ -1,08, 0,74]	95	0,36	0,72
орально	5 и 48	0,3–5	2	20 / 20	-0,09 [ -0,48, 0,30]	68	0,44	0,66
перед и в темную фазу	1–12	0,05–10	7	105 / 104	-0,47 [ -1,15, 0,21]	93	1,35	0,18
только молодые крысы (2–3 мес)	1–12	0,05–10	5	76 / 76	-1,38 [ -2,26, -0,50]	87	3,08	0,002
ТТГ (мМЕ/л)
≤1,5 мг/кг/день	1–80	0,05–1	7	123 / 123	0,00 [ -0,02, 0,03]	73	0,08	0,94
≥3 мг/кг/день	1 и 4	3 и 10	2	22 / 22	-0,28 [ -0,59, 0,04]	42	1,74	0,08
подкожно	1 и 12	0,125–10	3	36 / 36	-0,03 [ -0,16, 0,10]	79	0,43	0,67
внутрибрюшинно	4 и 8	0,05–3	3	42 / 42	-0,01 [ -0,05, 0,03]	83	0,49	0,62
орально	4–80	0,3–1,5	3	67 / 67	-0,03 [ -0,06, 0,00]	50	1,70	0,09
перед и в темную фазу	1–80	0,05–10	6	84 / 84	-0,01 [ -0,04, 0,02]	80	0,46	0,64
только молодые крысы (1,5–2 мес)	1–12	0,125–10	5	87 / 87	-0,02 [ -0,11, 0,07]	69	0,42	0,67
Кортикостерон (нг/мл)
≤1 мг/кг/день	1–48	0,04–1	8	181 / 181	19,78 [ -9,50, 49,06]	84	1,32	0,19
3-10 мг/кг/день	1–6	3,5–10	7	53 / 53	-39,60 [ -61,00, -18,20]	98	3,63	0,0003
подкожно	1–4	0,08–4,5	4	31 / 31	-10,12 [ -39,08, 18,84]	95	0,68	0,49
внутрибрюшинно	2–6	0,5–10	5	75 / 75	-9,80 [ -23,98, 4,37]	90	1,36	0,18
орально	1–48	0,04–5	7	128 / 128	-46,42 [ -117,86, 25,02]	95	1,27	0,20
перед и в темную фазу	1–6	0,5–10	3	60 / 60	-12,53 [ -32,42, 7,36]	70	1,23	0,22
только молодые крысы (1.5-4 мес)	1–12	0,5–10	6	101 / 101	-8,53 [ -63,13, 46,07]	88	0,31	0,76
возрастные крысы (6-18 мес)	1–48	0,04–5	4	50 / 50	-55,99 [ -152,16, 40,19]	97	1,14	0,25

**Figure:**
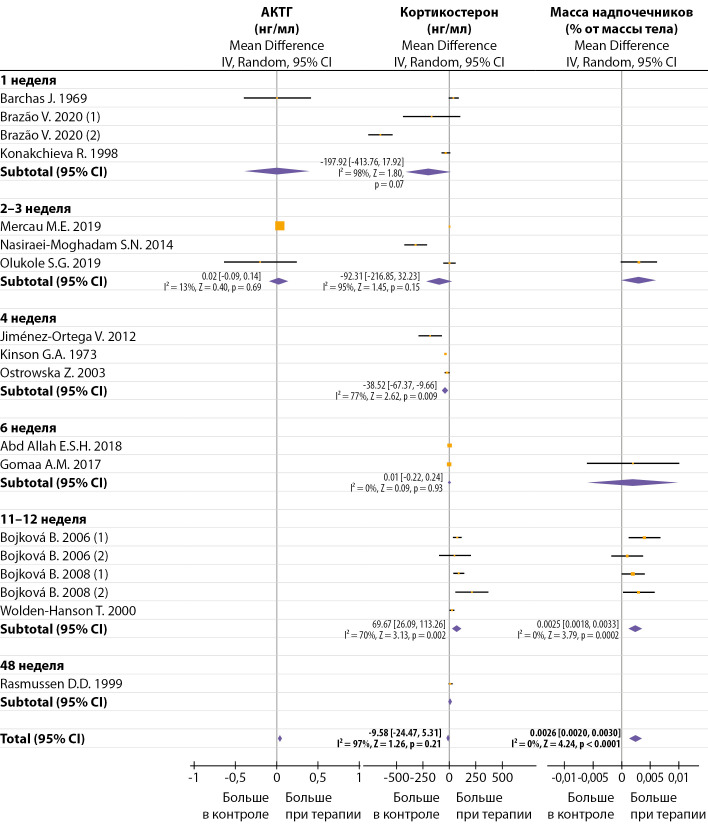
Рисунок 5. Влияние продолжительной терапии мелатонином на уровень циркулирующих АКТГ, кортикостерона и массу надпочечников. Без дополнительной стандартизации по дозе и способу введения.

**Figure:**
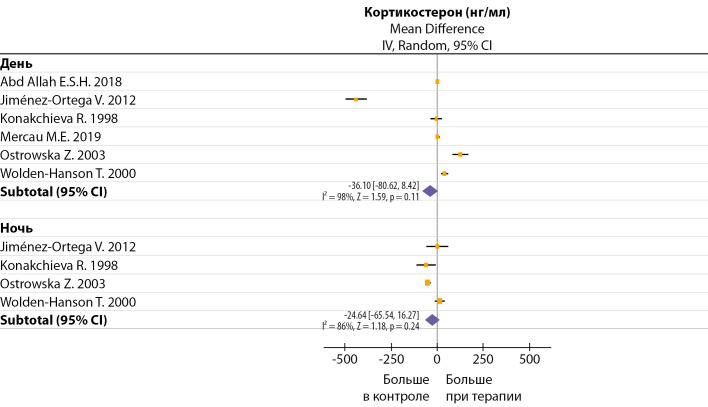
Рисунок 6. Влияние продолжительной терапии мелатонином на изменение дневных и ночных значений кортикостерона. Без дополнительной стандартизации по дозе и способу введения.

## ЗАКЛЮЧЕНИЕ

В итоге не было получено убедительных данных об изменении уровня ТТГ и ТГ после терапии мелатонином без учета дозы и сроков терапии, но наблюдалась тенденция снижения ТТГ, Т3 и Т4 при более высоких дозах мелатонина, однако работ слишком мало для окончательного вывода. Начало терапии было ассоциировано со снижением активности ТГ, после 7 недель терапии активность ТГ незначительно возрастала. Максимальную чувствительность к экзогенному мелатонину показал Т4, что свидетельствует о влиянии мелатонина на секреторную функцию ЩЖ.

По результатам нашего метаанализа, эффект мелатонина на уровень КС зависит от дозы и продолжительности терапии. Снижение КС было ассоциировано с терапией продолжительностью не более 4 недель и с высокими дозами мелатонина. Повышение КС наблюдалось при длительной терапии. Кроме того, мелатонин увеличивал относительный вес надпочечников. К настоящему времени плохо изучена секреция КС при использовании мелатонина в дозах 10 мг/кг и более.

Интересно отметить, что ранее проведенный нами метаанализ показал: первые недели терапии мелатонином и более высокие дозы гормона ассоциированы с увеличением уровня циркулирующей глюкозы, триглицеридов, инсулина у крыс, содержащихся в стандартных условиях [75]. Результаты настоящего и предыдущего [75] метаанализов свидетельствуют о том, что воздействие мелатонина на углеводный и липидный обмен может осуществляться в том числе и через модулирование уровня ТГ и КС.

Следует учесть, что при каких-либо возмущающих воздействиях эффект мелатонина на параметры метаболизма может изменяться. Например, при стандартной диете мелатонин практически не влияет на липидный профиль, однако при высокофруктозной, высокожировой и высокохолестериновой диете мелатонин эффективно снижает уровень триглицеридов и холестерина [75]. Это объясняется тем, что, с одной стороны, высококалорийные диеты могут изменять экспрессию и аффинность рецепторов, чувствительных к мелатонину; а с другой — мелатонин нивелирует окислительный стресс, вызванный диетами.

## ДОПОЛНИТЕЛЬНАЯ ИНФОРМАЦИЯ

Источники финансирования. Финансирование из средств государственного задания №056-00119-22-00.

Конфликт интересов. Авторы декларируют отсутствие конфликта интересов.

Вклад авторов. Кузьменко Н.В, Плисс М.Г. — концепция,подбор литературы, проведение метаанализа; Кузьменко Н.В., Цырлин В.А. — написание текста; Кузьменко Н.В. — иллюстрации. Все авторы одобрили финальную версию статьи перед публикацией, выразили согласие нести ответственность за все аспекты работы, подразумевающую надлежащее изучение и решение вопросов, связанных с точностью или добросовестностью любой части работы.
